# Supplementary material for: Polymerizable rotaxane hydrogels for three-dimensional printing fabrication of wearable sensors
Source: Nat Commun. 2023 Mar 10;14:1331. doi: 10.1038/s41467-023-36920-3 (PMC10006079; doi:10.1038/s41467-023-36920-3)
Supplement: Supplementary file 1 — Supplementary Information [file 41467_2023_36920_MOESM1_ESM.pdf]

Supplementary Information

Polymerizable rotaxane hydrogels for three-dimensional printing fabrication of wearable sensors

Xueru Xiong, Yunhua Chen, Zhenxing Wang, Huan Liu, Mengqi Le, Caihong Lin, Gang Wu, Lin Wang*, Xuetao Shi*, Yong-Guang Jia*, Yanli Zhao*

*Corresponding authors. Email: wanglin3@scut.edu.cn (L.W.); shxt@scut.edu.cn (X.S.); ygjia@scut.edu.cn (Y.G.J.); zhaoyanli@ntu.edu.sg (Y.Z.)

This PDF file includes:

Supplementary Figs. 1 to 22

Supplementary Table 1

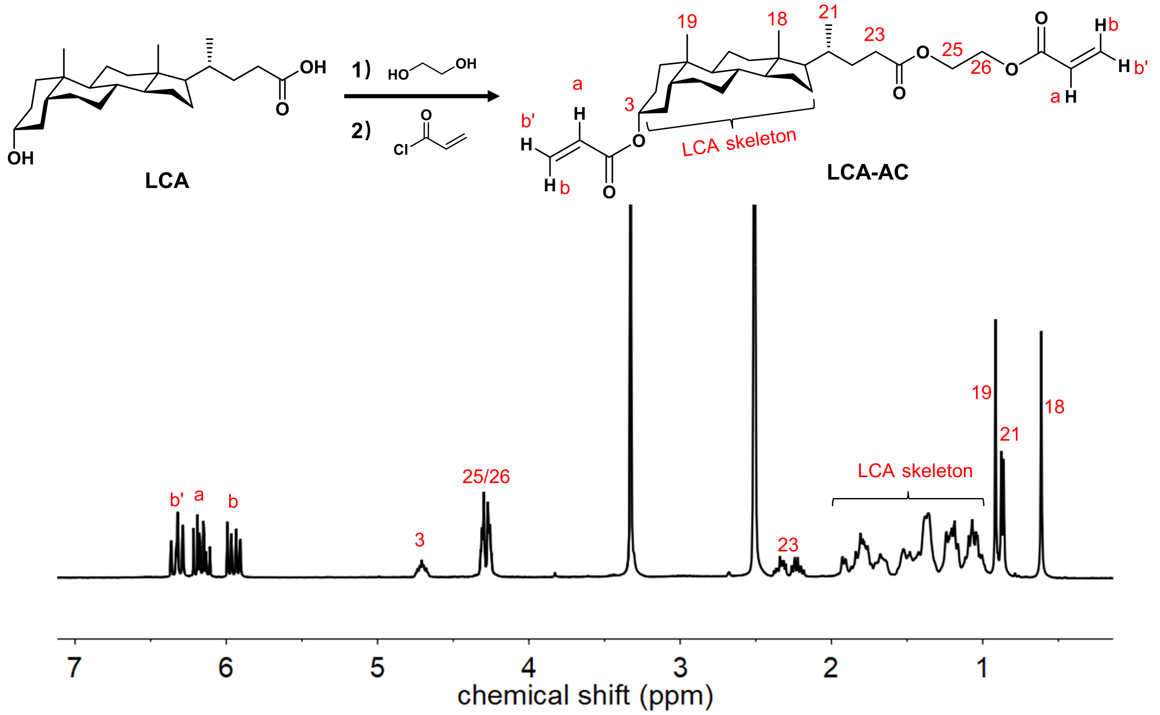

Supplementary Fig. 1. Synthesis route of LCA-AC and its ^{1}H NMR spectrum obtained in DMSO-d$_6$ as well as the assignments of peaks.

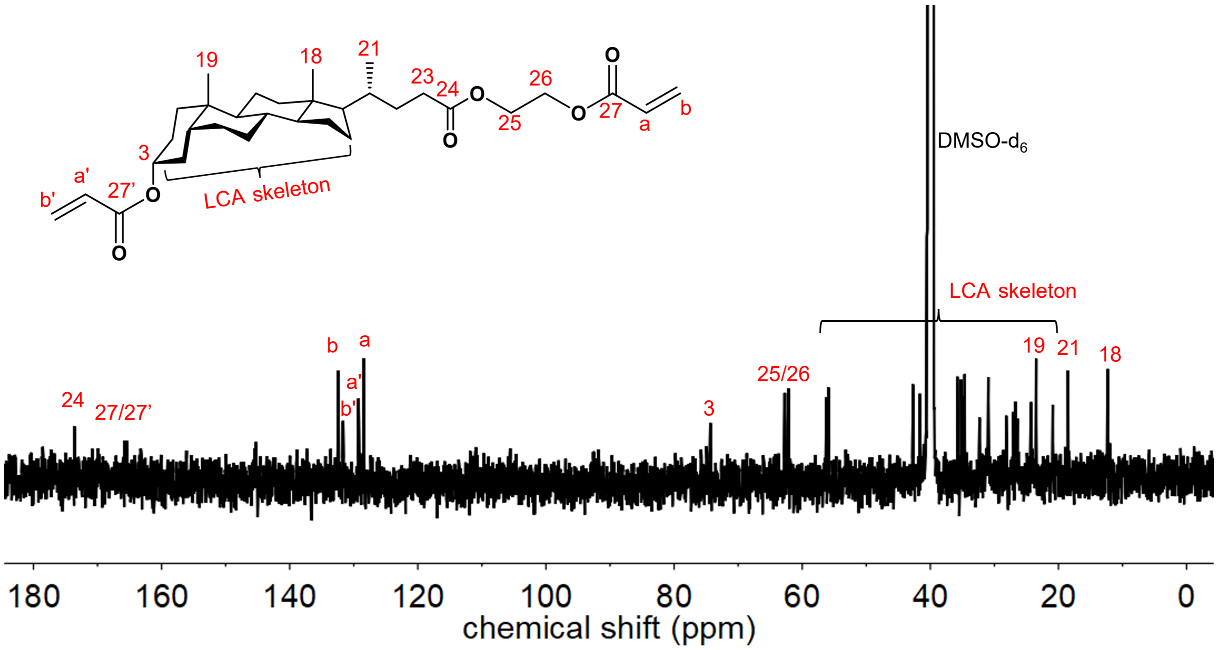

Supplementary Fig. 2. ^{13}C NMR spectrum of LCA-AC obtained in DMSO-d_6 and the assignments of peaks.

Acquisition Parameter

Source Type	ESI	Ion Polarity	Positive	Set Nebulizer	0.6 Bar
Focus	Active	Set Capillary	3500 V	Set Dry Heater	180 °C
Scan Begin	50 m/z	Set End Plate Offset	-500 V	Set Dry Gas	4.0 l/min
Scan End	1500 m/z	Set Charging Voltage	0 V	Set Divert Valve	Waste
		Set Corona	0 nA	Set APCI Heater	0 °C

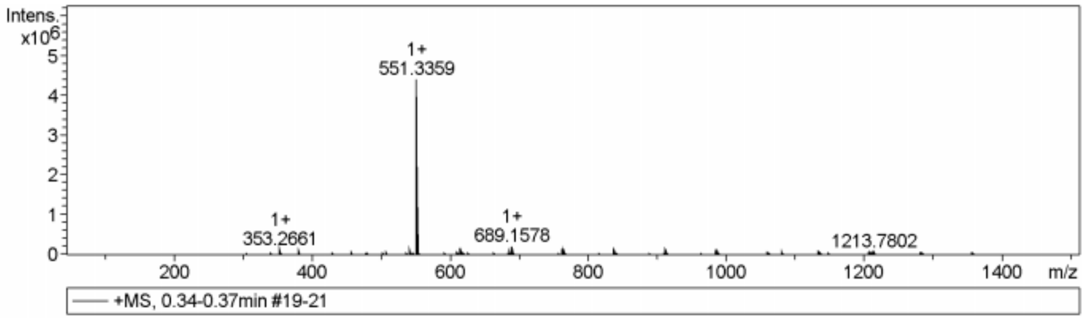

#	m/z	I	I %
1	353.2661	121110	2.8
2	381.2973	81511	1.9
3	541.1202	108733	2.5
4	551.3359	4395613	100.0
5	552.3388	1365252	31.1
6	553.3407	248691	5.7
7	615.1392	174537	4.0
8	616.1395	102343	2.3
9	617.1374	79083	1.8
10	685.4351	82173	1.9
11	689.1578	211996	4.8
12	690.1584	134668	3.1
13	691.1561	109614	2.5
14	763.1766	161506	3.7
15	764.1772	119152	2.7
16	765.1755	95956	2.2
17	837.1950	128005	2.9
18	838.1961	103395	2.4
19	839.1941	87623	2.0
20	911.2139	106331	2.4
21	912.2145	91365	2.1
22	913.2131	83984	1.9
23	985.2321	88440	2.0
24	986.2328	82975	1.9
25	987.2319	79595	1.8
26	1059.2511	74525	1.7
27	1060.2521	73998	1.7
28	1061.2507	69812	1.6
29	1079.6783	84700	1.9
30	1213.7802	66590	1.5

Supplementary Fig. 3. ESI-HRMS spectrum of LCA-AC. Calculated for $C_{32}H_{48}NaO_6$, $[M+Na]^+$ 551.3349. Found: 551.3359.

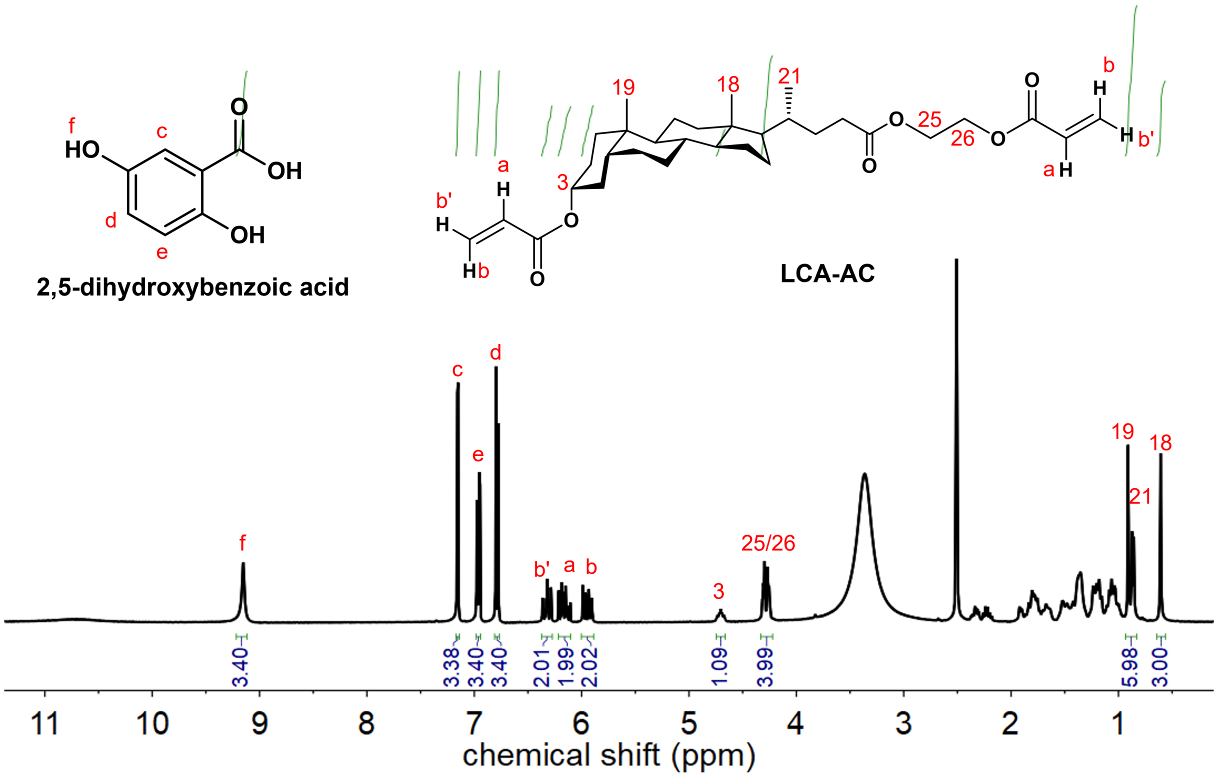

Supplementary Fig. 4. ^{1}H NMR spectrum of LCA-AC (5.08 mg) mixed with internal standard 2,5-dihydroxybenzoic acid (5.03 mg) in DMSO-d_6 and the assignments of the related peaks. The purity of LCA-AC was quantified based on the integration ratio of peaks c, d or e to 18 and the content of LCA-AC was calculated to be ca. 98.9%.

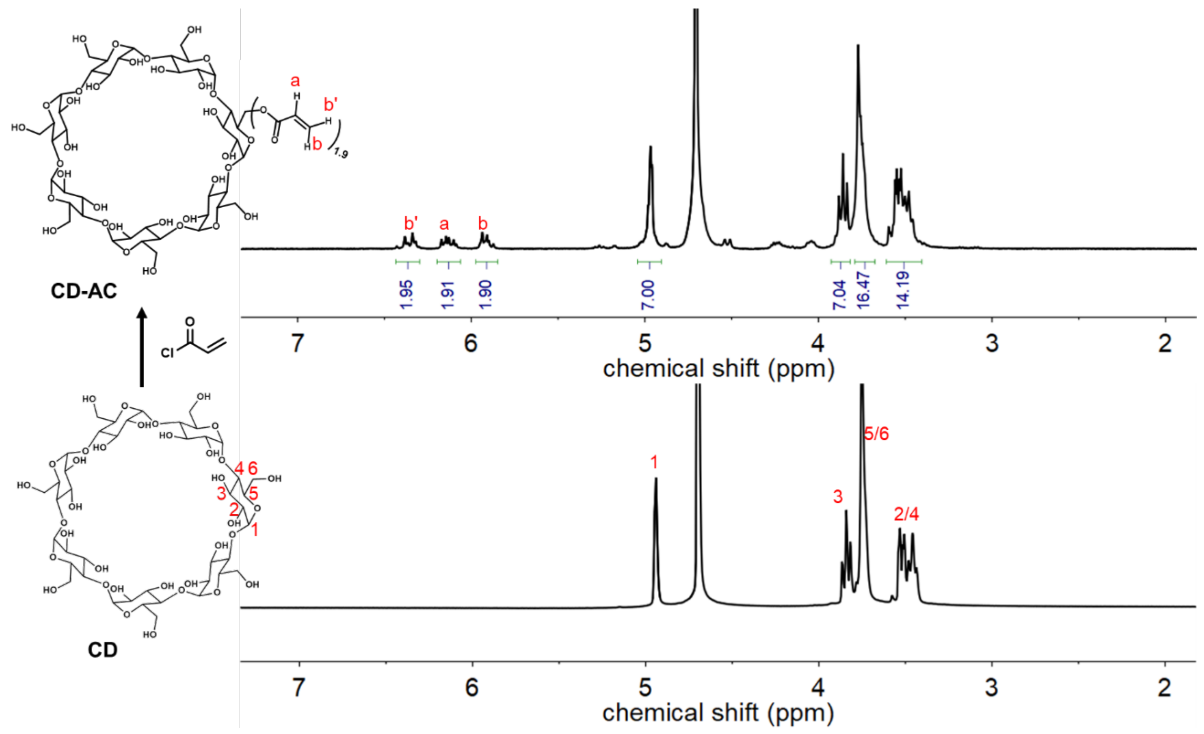

Supplementary Fig. 5. Synthesis route of CD-AC and its ^{1}H NMR spectrum in D_2O and the assignments of peaks. Acrylate units on each CD-AC were estimated to be ca. 1.91 based on the integration ratio of peaks a, b or b' to peak 1.

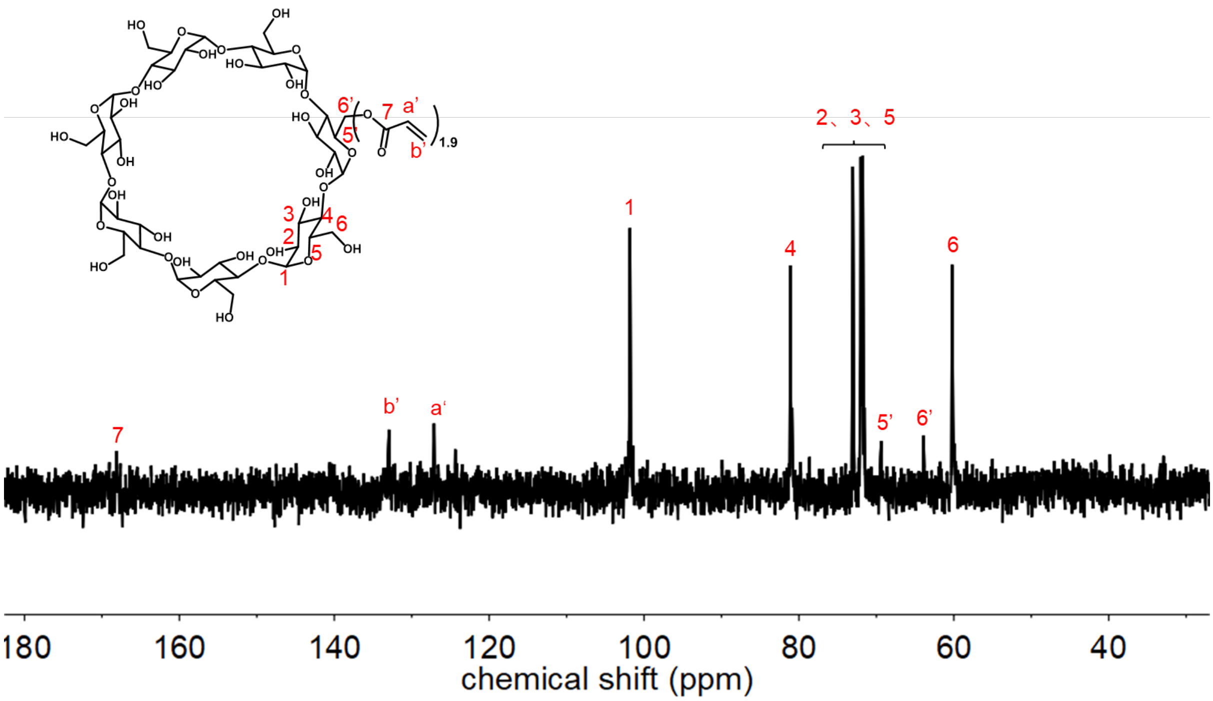

Supplementary Fig. 6. ^{13}C NMR spectrum of CD-AC in D_2O and the assignments of peaks.

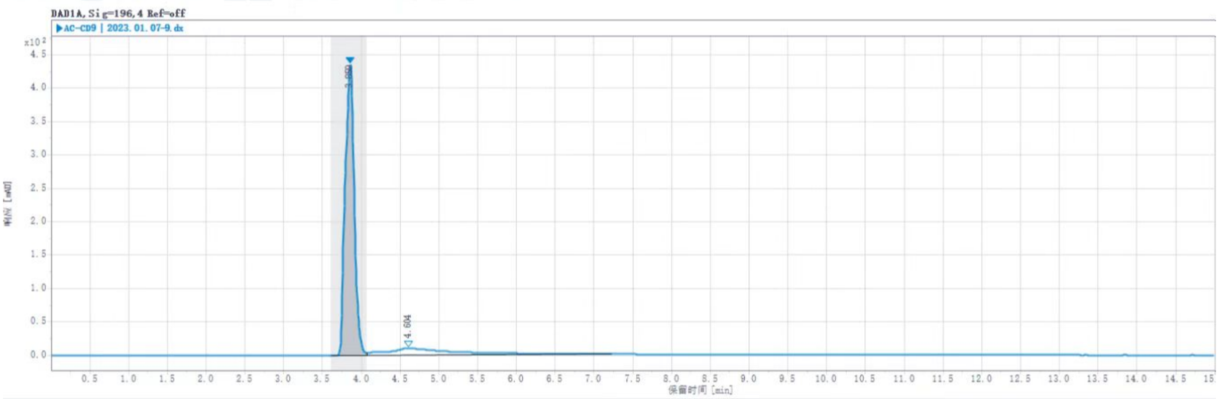

进样结果

峰 汇总

#	名称	信号说明	RT (min)	峰面积 (mAU·s)	峰面积 %	峰高 (mAU)	峰高%	含量	浓度	开始时间 (min)	结束时间 (min)
1		DAD1A,Sig=196,4 Ref=off	3.859	3565.261	83.800	435.676	97.64			3.614	4.074
2		DAD1A,Sig=196,4 Ref=off	4.604	689.208	16.200	10.508	2.36			4.074	7.226

Supplementary Fig. 7. HPLC result of CD-AC further confirming that the purity of diacrylated β-CD in mixture is about 83.8%.

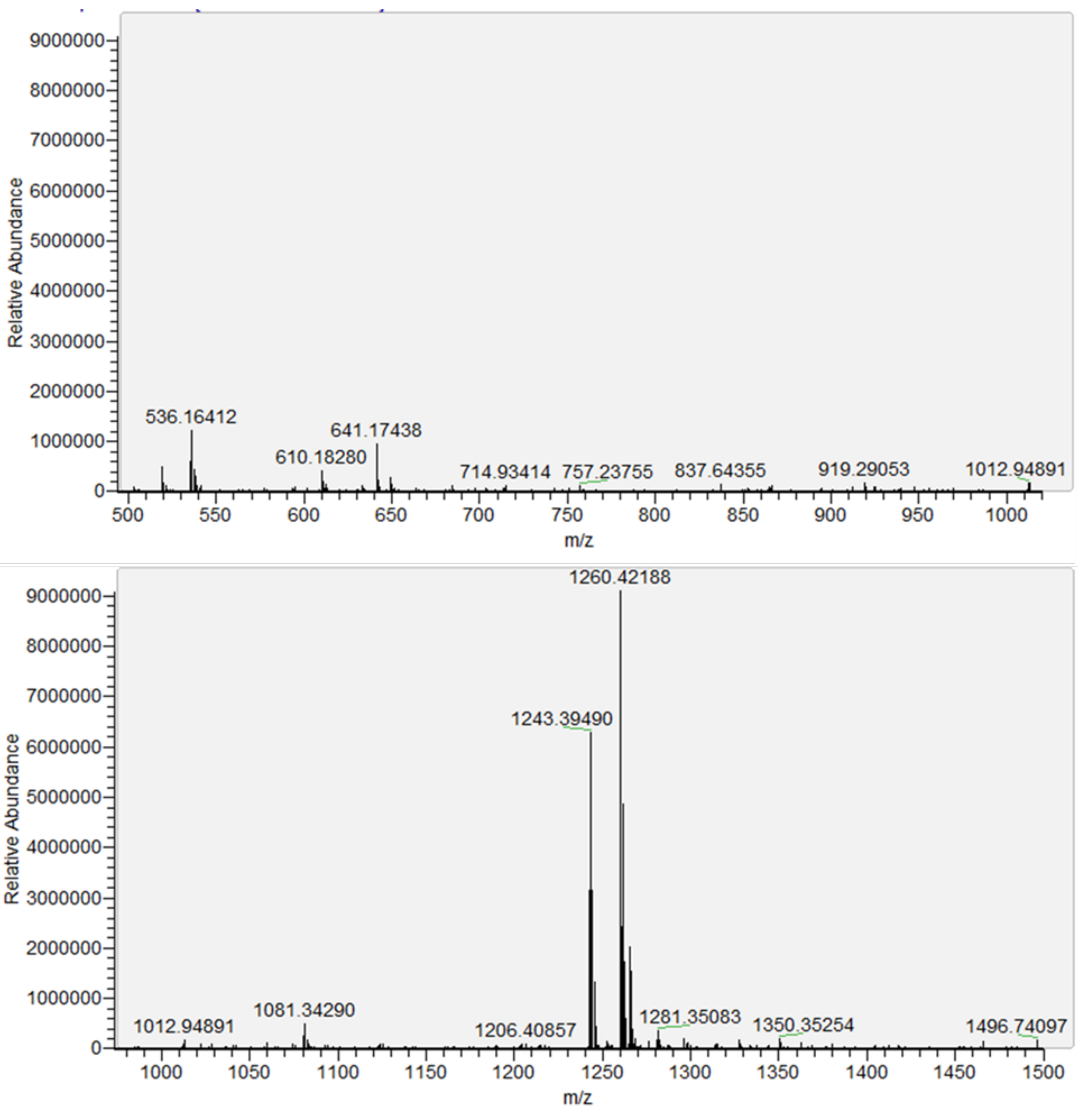

Supplementary Fig. 8. ESI-HRMS spectrum of CD-AC indicating that the most abundant component is the diacrylated β-CD. Calculated for diacrylated β-CD: $C_{48}H_{75}O_{37}$, $[M+H]^+$ 1243.3987. Found: 1243.3949.

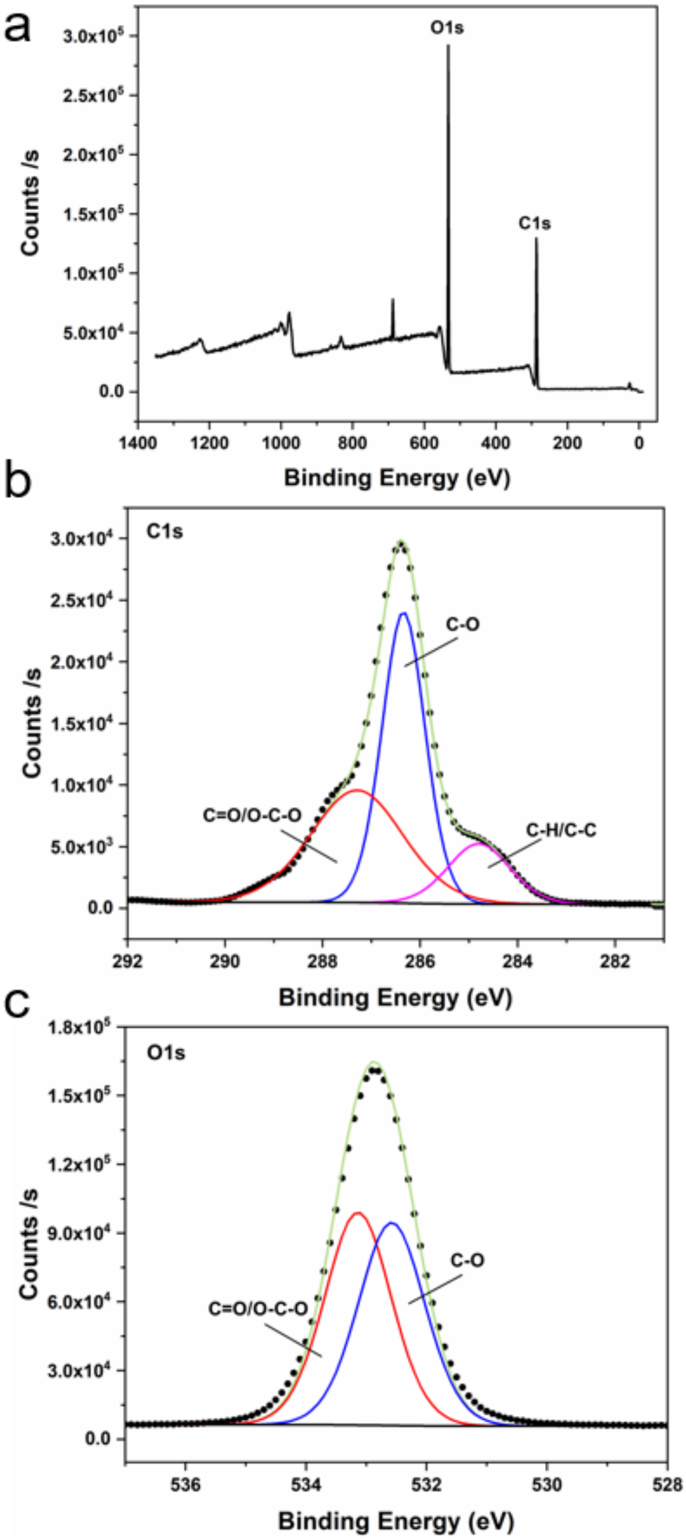

Supplementary Fig. 9. XPS spectra. (a) Wide scan XPS spectrum of CD-AC, and (b) C 1s and (c) O 1s XPS spectra of CD-AC.

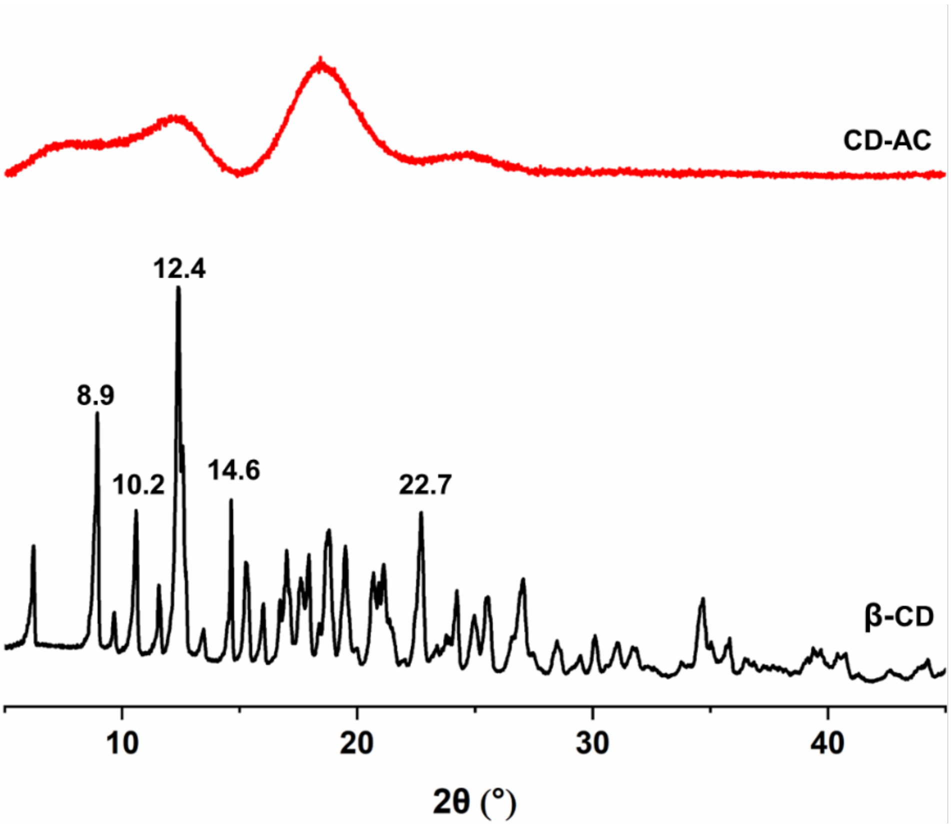

Supplementary Fig. 10. Powder XRD patterns of CD-AC and β-CD, indicating CD-AC becomes an amorphous state compared with β-CD.

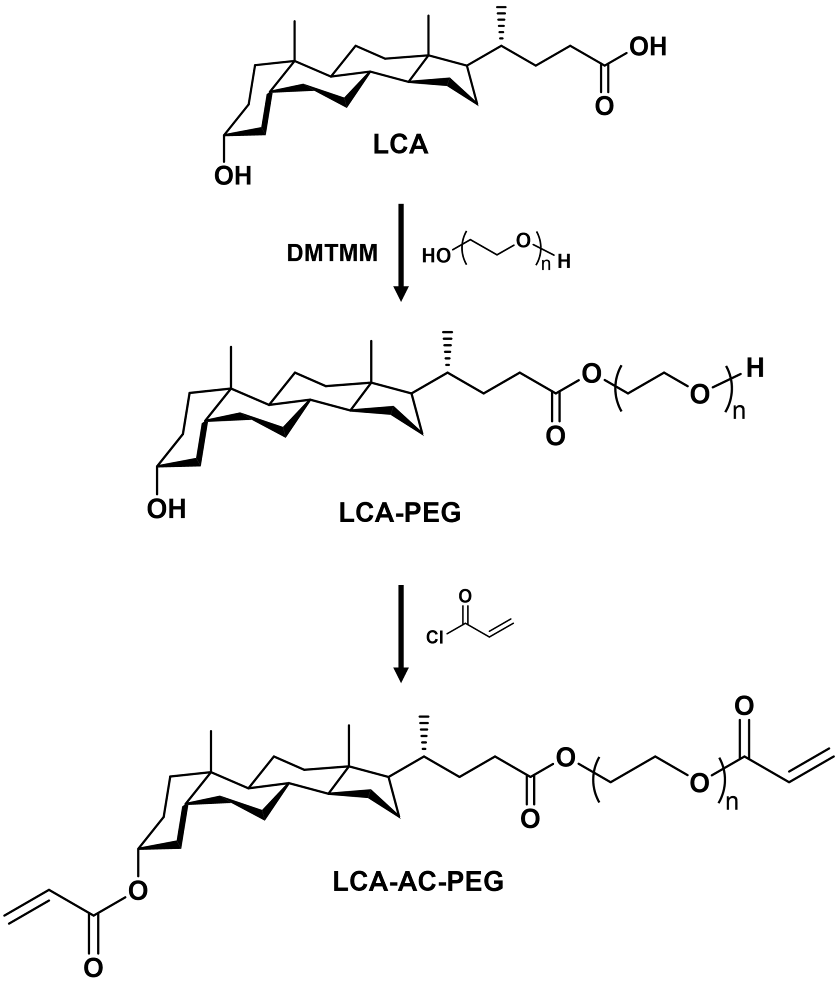

Supplementary Fig. 11. Synthesis route of LCA-AC-PEG.

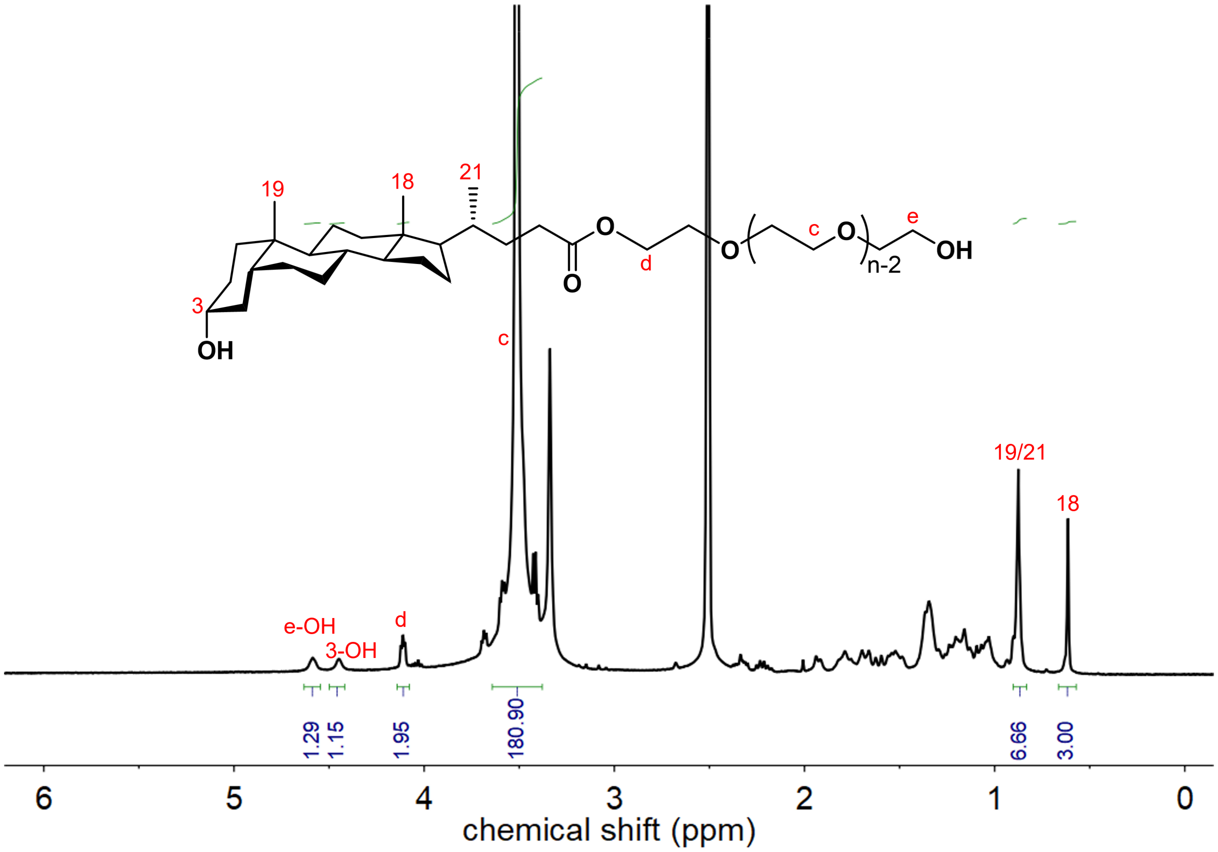

Supplementary Fig. 12. ^{1}H NMR spectrum of LCA-PEG in DMSO-d$_6$ and the assignment of peaks.

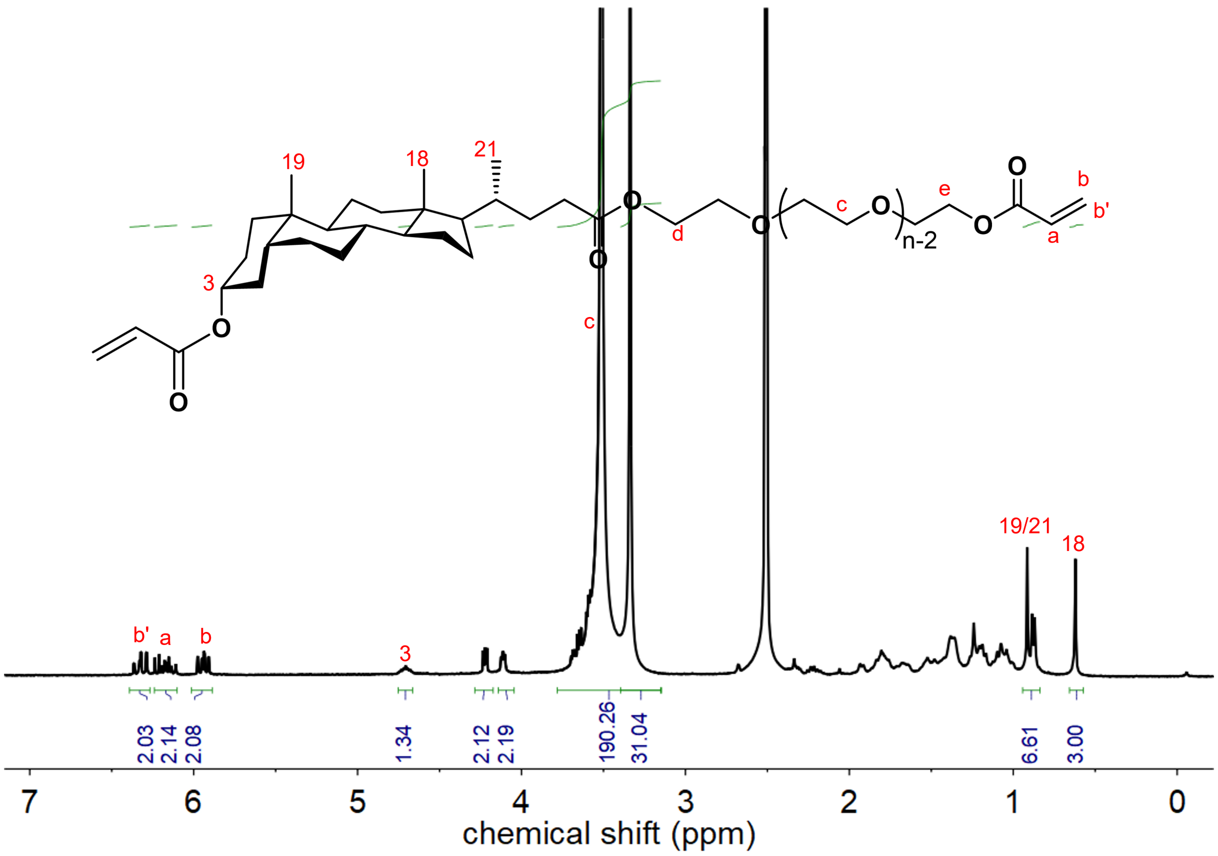

Supplementary Fig. 13. ^{1}H NMR spectrum of LCA-AC-PEG in DMSO-d_6 and the assignment of peaks.

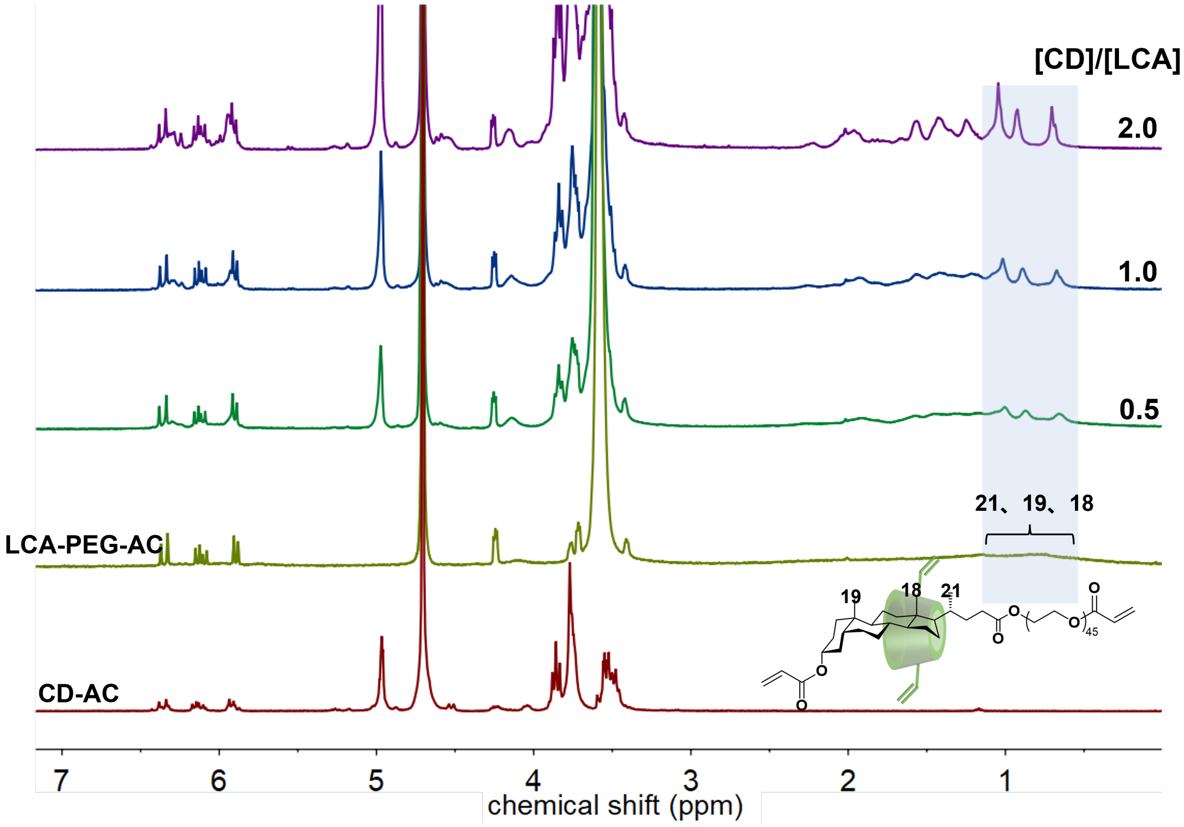

Supplementary Fig. 14. ^{1}H NMR spectra of LCA-AC-PEG (D_2O) in the presence of different equivalents of CD-AC. Signals of the three methyl protons on the LCA moieties all shift downfield and become sharper, indicating the formation of host-guest complex.

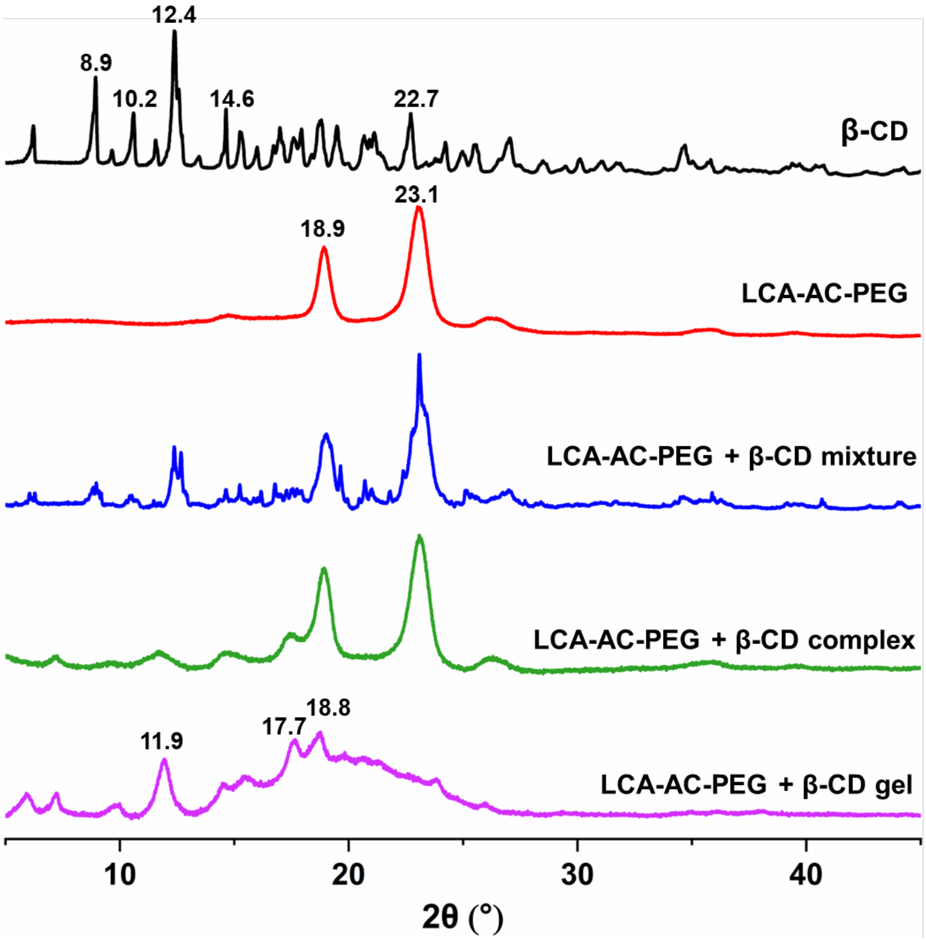

Supplementary Fig. 15. Powder XRD patterns of β-CD, LCA-AC-PEG, the mixture of β-CD with LCA-AC-PEG (1/1 mol.), β-CD/LCA-AC-PEG host-guest complex (1/1 mol.) and the polyacrylamide hydrogels crosslinked by β-CD/LCA-AC-PEG host-guest complex (1/1 mol.).

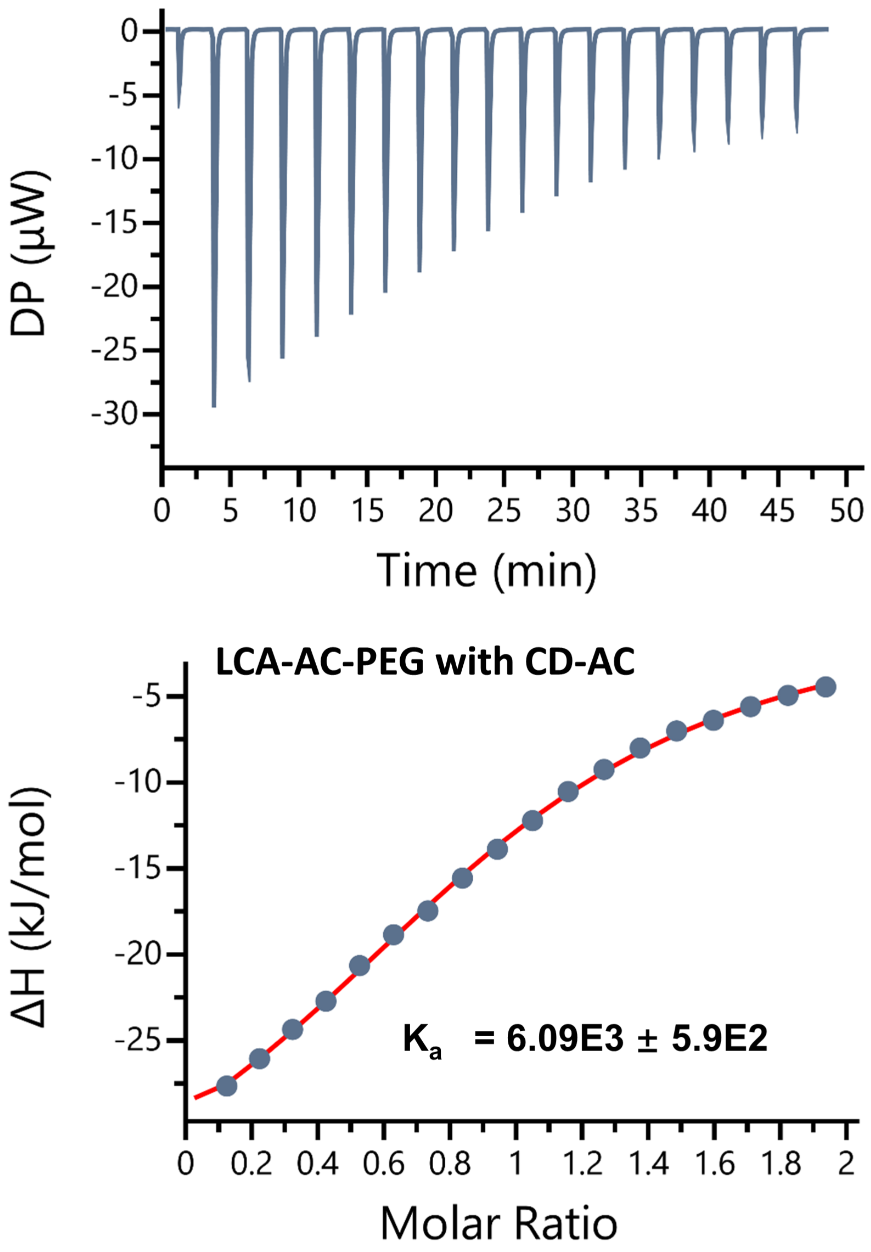

Supplementary Fig. 16. Apparent reaction heat in calorimetric titrations of CD-AC aqueous solution (5.0 mM) injecting into LCA-AC-PEG aqueous solution (0.5 mM) at 25 °C and their typical isothermal titration calorimetry (ITC) fitting curves.

Supplementary Table 1. Recipe of precursor solutions for the synthesis of conductive hydrogels. The total volume of the solution in each group was 1 mL.

Hydrogels	Am (g)	PR/Am (mol%)	CD-AC/Am (mol%)	MBA/Am (mol%)	EG/Water (vol.)	ChCl (mmol)	I_{2959}/Am (mol%)
**PR-Gel** (20 w/v%)	0.2	0.5	0	0	1/1	3	1
PR-Gel (30 w/v%)	0.3	0.5	0	0			
PR-Gel (40 w/v%)	0.4	0.5	0	0			
**CD-Gel**	0.4	0	0.5	0			
**MBA-Gel**	0.4	0	0	0.5			

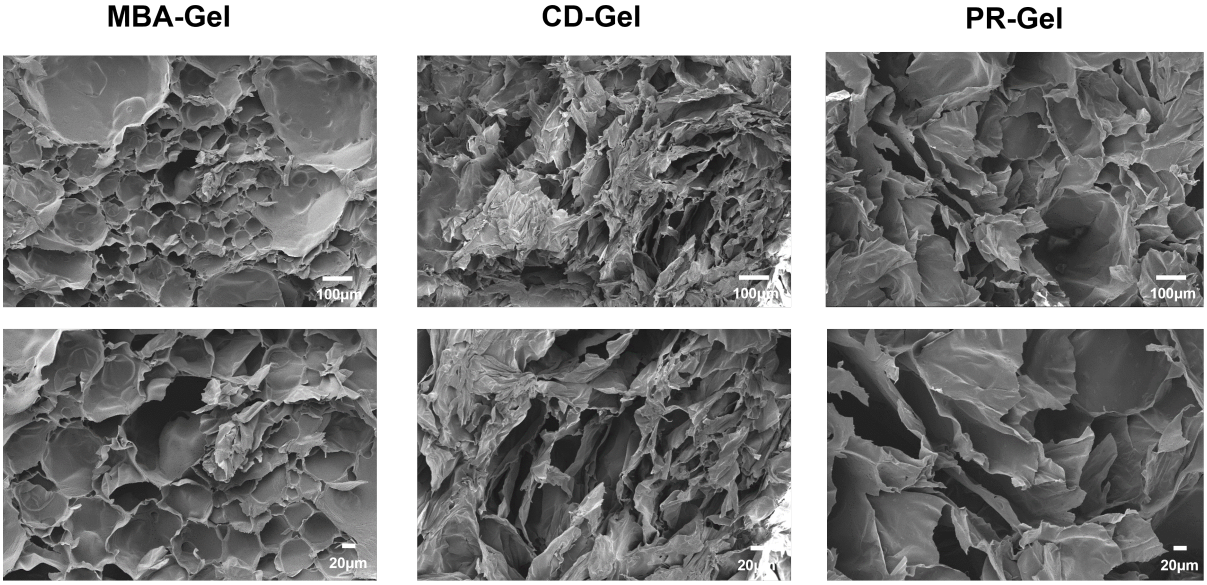

Supplementary Fig. 17. SEM images of lyophilized MBA-Gel, CD-Gel and PR-Gel with a concentration of Am 40 w/v% and their corresponding enlarged images.

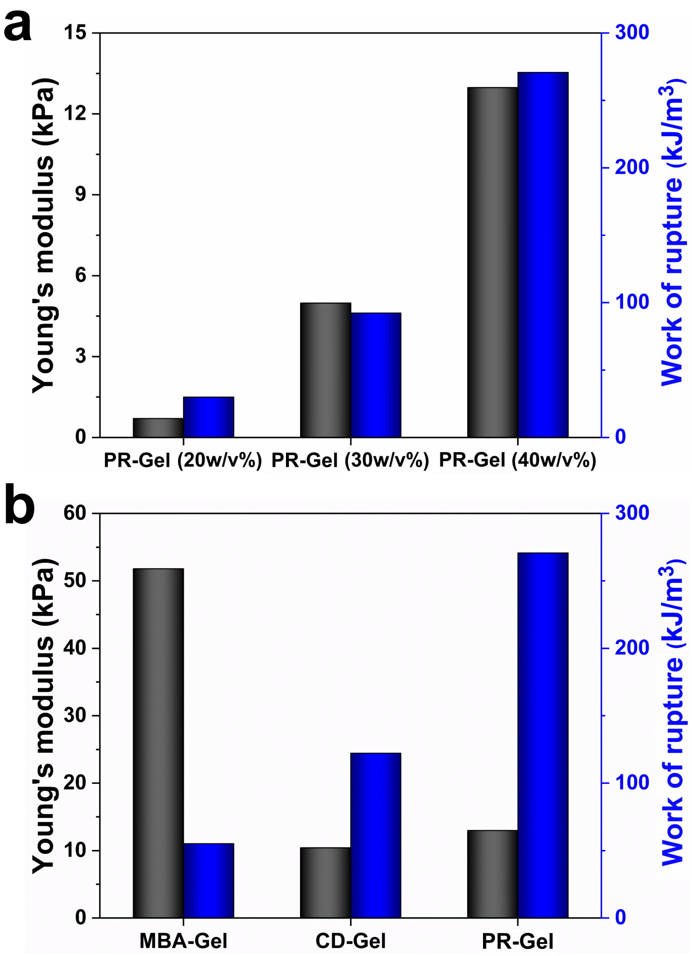

Supplementary Fig. 18. Young's modulus and work of rupture. (a) PR-Gel with different concentrations of Am and (b) gels with different crosslinkers at a concentration of Am 40 w/v%.

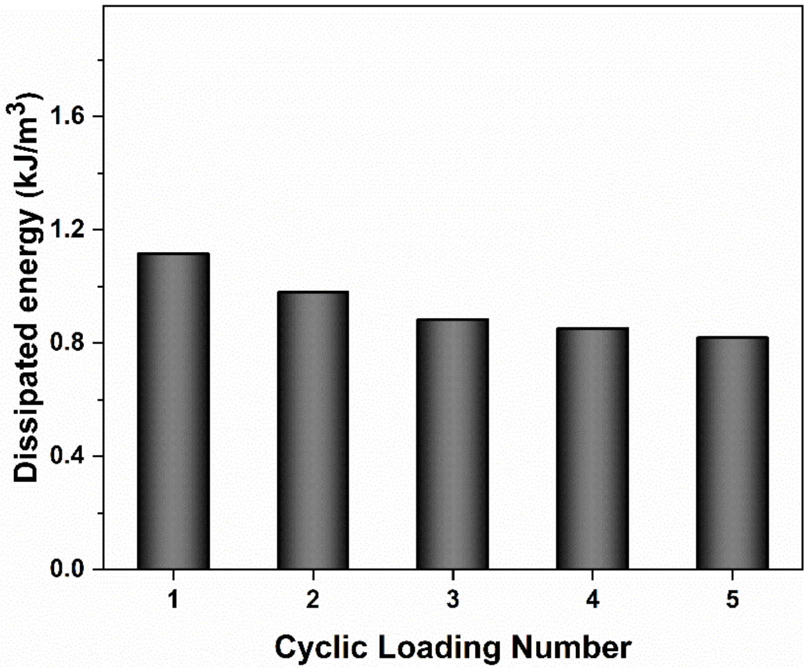

Supplementary Fig. 19. Dissipation energy of PR-Gel (40 w/v%) in 5 consecutive cycles of tensile at 300% strain.

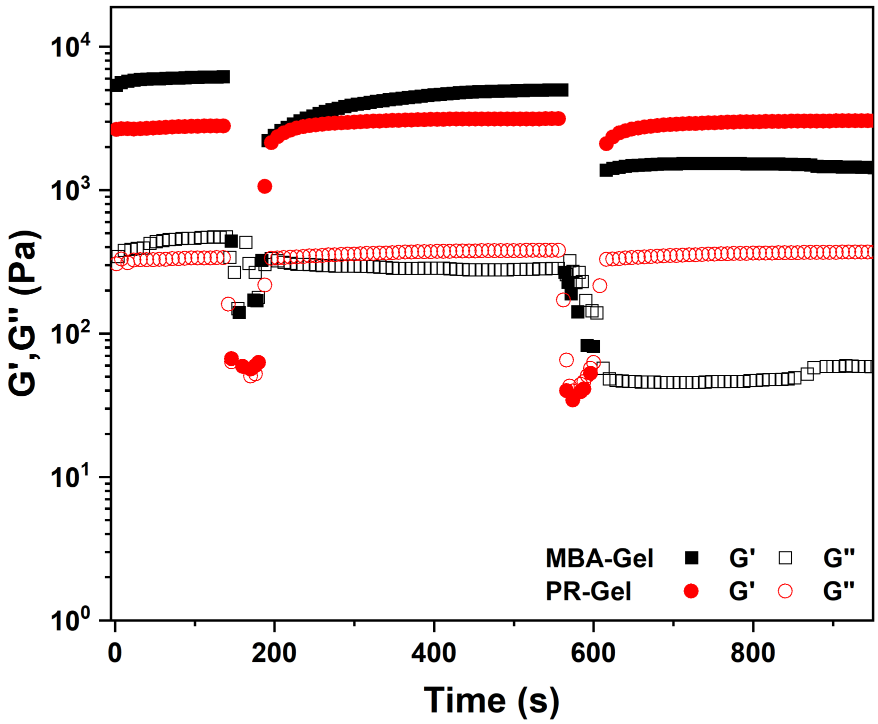

Supplementary Fig. 20. Storage modulus (G') and loss modulus (G'') of PR-Gel and MBA-Gel with a concentration of Am 40 w/v% at alternate strains between subtle strain (1%) and large strain (500%).

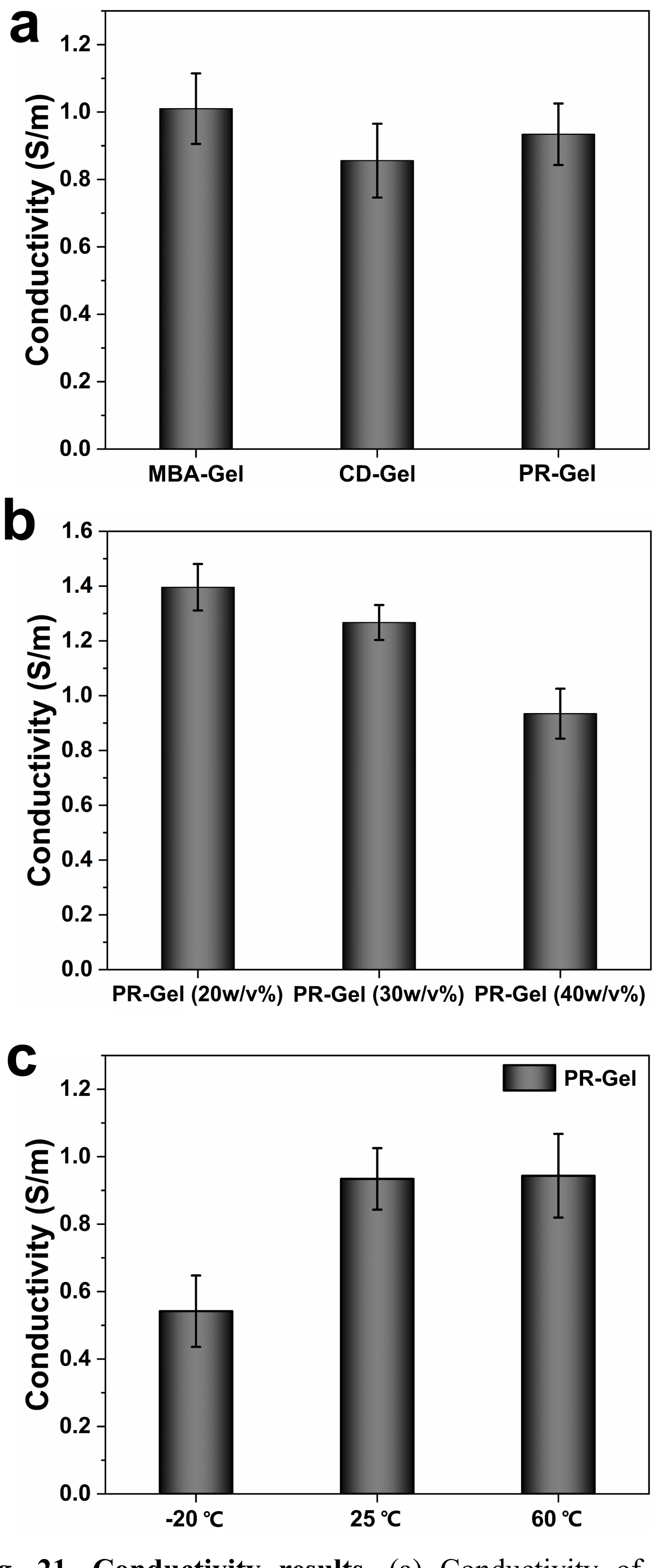

Supplementary Fig. 21. Conductivity results. (a) Conductivity of gels with different crosslinkers. (b) Conductivity of PR-Gel at different concentrations. (c) Conductivity of PR-Gel (40 w/v%) at different temperatures.

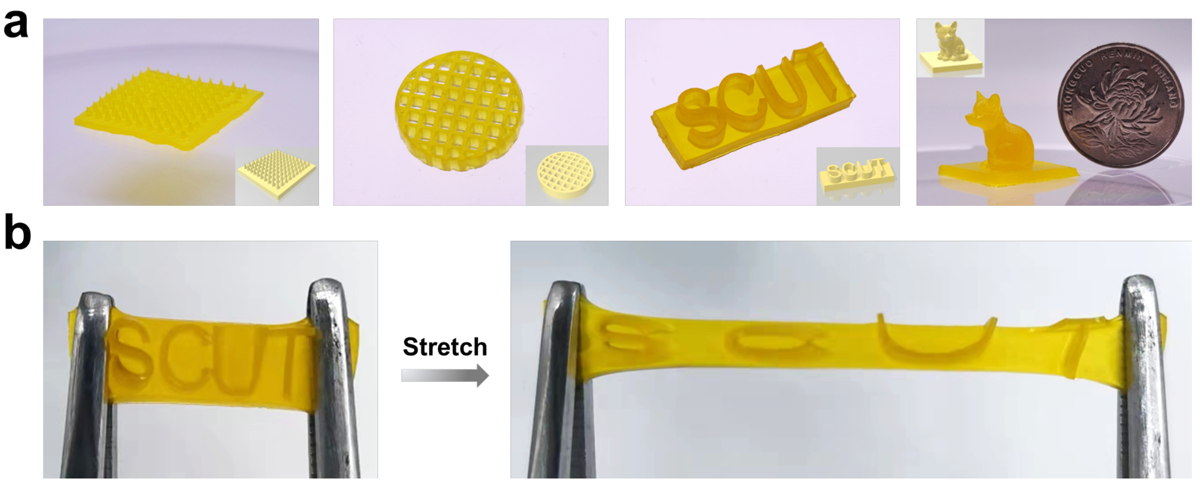

Supplementary Fig. 22. 3D printed PR-Gel. (a) PR-Gel (40 w/v%) based skeletons of various shapes fabricated with digital light processing (DLP) technology, and tartrazine as a dye. (b) Photographs of 3D printed PR-Gel (40 w/v%) before and after stretching.
