# Supplementary material for: Polymerizable rotaxane hydrogels for three-dimensional printing fabrication of wearable sensors
Source: Nat Commun. 2023 Mar 10;14:1331. doi: 10.1038/s41467-023-36920-3 (PMC10006079; doi:10.1038/s41467-023-36920-3)
Supplement: Supplementary file 3 — Description of Additional Supplementary Information [file 41467_2023_36920_MOESM3_ESM.pdf]

Description of Additional Supplementary Information

Title: Supplementary Movie 1

Description: Compression toughness and recovery performance of PR-Gel.

Title: Supplementary Movie 2

Description: Puncture resistance of PR-Gel.
